# Cholecystectomy promotes the development of colorectal cancer by the alternation of bile acid metabolism and the gut microbiota

**DOI:** 10.3389/fmed.2022.1000563

**Published:** 2022-09-23

**Authors:** Xi Jiang, Zhongxiu Jiang, Qi Cheng, Wei Sun, Min Jiang, Yan Sun

**Affiliations:** ^1^Department of Cardiology, Shengjing Hospital of China Medical University, Shenyang, Liaoning, China; ^2^Department of Oncology, Shengjing Hospital of China Medical University, Shenyang, Liaoning, China; ^3^Department of Pediatrics, Shengjing Hospital of China Medical University, Shenyang, Liaoning, China; ^4^Department of Ultrasound, Shengjing Hospital of China Medical University, Shenyang, Liaoning, China; ^5^Department of Gastroenterology, First Affiliated Hospital of China Medical University, Shenyang, Liaoning, China; ^6^Department of Gastroenterology, Shengjing Hospital of China Medical University, Shenyang, Liaoning, China

**Keywords:** colorectal cancer, cholecystectomy, bile acid metabolism, gut microbiota, development

## Abstract

The incidence and mortality of colorectal cancer (CRC) have been markedly increasing worldwide, causing a tremendous burden to the healthcare system. Therefore, it is crucial to investigate the risk factors and pathogenesis of CRC. Cholecystectomy is a gold standard procedure for treating symptomatic cholelithiasis and gallstone diseases. The rhythm of bile acids entering the intestine is altered after cholecystectomy, which leads to metabolic disorders. Nonetheless, emerging evidence suggests that cholecystectomy might be associated with the development of CRC. It has been reported that alterations in bile acid metabolism and gut microbiota are the two main reasons. However, the potential mechanisms still need to be elucidated. In this review, we mainly discussed how bile acid metabolism, gut microbiota, and the interaction between the two factors influence the development of CRC. Subsequently, we summarized the underlying mechanisms of the alterations in bile acid metabolism after cholecystectomy including cellular level, molecular level, and signaling pathways. The potential mechanisms of the alterations on gut microbiota contain an imbalance of bile acid metabolism, cellular immune abnormality, acid-base imbalance, activation of cancer-related pathways, and induction of toxin, inflammation, and oxidative stress.

## Introduction

Colorectal cancer (CRC) is the third most malignancy worldwide for humans (1–3). The incidence and mortality of CRC are terrifyingly high. CRC accounts for over 9% of all cancers incidence (4). It has been estimated that approximately 53,200 deaths projected in 2020 (5) and 3.2 million new CRC cases projected in 2040 (6). CRC exerts a significant geographic difference, more common in the western developed countries (7–9). The incidence rate was 10-fold higher in the highest rate countries than that in the lowest rate countries (10). The incidence of CRC in China is 23.3 per 100,000 (11). In addition, the prevalence of CRC has been growing in the young individuals (12–14), contributing to substantial social and economic burden to the healthcare system (2, 6). Therefore, it is essential to explore the risk factors and pathogenesis of CRC.

Several etiologies have been implicated in the pathogenesis of CRC, including genetic susceptibility and environmental factors, such as consumption of tobacco and alcohol, inflammatory bowel disease (IBD), adenomatous polyps, family history, unhealthy diet, physical inactivity and obesity (8, 15–17). Cholecystectomy is a standard procedure for treatment of symptomatic cholelithiasis and gallstone diseases. The number of this procedure has been increasing. It has been reported that approximately 800,000 cases of cholecystectomy are performed in the United States per year, and the number is also growing in China (18, 19). In the past, cholecystectomy was deemed to be almost harmless. Nevertheless, an increasing body of evidence suggests that cholecystectomy might be associated with the development of CRC (20–25). Alterations of bile acid metabolism and gut microbiota have been demonstrated to play significant roles in CRC. However, the potential specific mechanisms are still unclear. Therefore, in the present study, we described the current knowledge on the association between cholecystectomy and CRC, and summarized the potential mechanisms.

## Epidemiology of colorectal cancer

CRC is the third most common cause of cancer-related mortality worldwide (2), and is also the second most common cause of cancer mortality in the United States (26). It has estimated that more than 1.8 million cases were diagnosed and 881,000 deaths occurred in 2018, accounting for 1 in 10 cancer cases and deaths (11). More recently, over 1.9 million new cases were reported in 2020 (27). Globally, CRC incidence and mortality vary widely across countries, according to GLOBOCAN 2020 data (28). The incidence of CRC is higher in males than in females, and the trend is younger in recent years (12–14). In general, the incidence of proximal colon tumors is the highest, while that of distal colon tumors is the lowest, which is more common in the elderly. The incidence rates increased by 1 and 2% each year among the 50–64 age group and under 50 years of age, respectively (26). The population of CRC patients as a whole is rapidly getting younger as the declining incidence in the older population coincides with the increasing incidence in the younger population, causing huge burden on the healthcare system. It has been well-acknowledged that CRC is associated with several risk factors such as smoking, unhealthy diet, alcohol abuse, physical inactivity and obesity (15, 16, 29). Nevertheless, the risk factors and pathogenesis of CRC still need to be further explored.

## Cholecystectomy

Cholecystectomy is the most common procedure performed in biliary surgery. In most cases, the procedure is relatively standardized and the long-term results after surgery are satisfactory. Cholecystectomy can be performed in two main ways: a laparoscopic or a classic open operation technique. Compared to the classic way, the laparoscopic cholecystectomy is a relatively minimally invasive surgical procedure and has basically replaced the open technique for cholecystectomies since the early 1990s (30). Several advantages have made it a popular procedure over the past few decades, including a short hospital stay, quick return to normal activities, and reduced pain after surgery, more acceptable cosmetic results, less morbidity and less mortality (31–33). The reported short-term complications include postoperative bleeding, biliary leakage biliary peritonitis, subhepatic effusion or subphrenic abscess, postoperative jaundice, postoperative pancreatitis, residual common bile duct stones, and gastrointestinal fistula, and the long-term complications include bile duct stricture, recurrent common bile duct stones, biliary bleeding, post-cholecystectomy syndrome, residual overgrown bile duct syndrome, and increased incidence of CRC.

## Clinical data and characteristics of colorectal cancer patients after cholecystectomy

A meta-analysis of 10 cohort studies described that there was an increased risk for colon cancer up to 30% higher than the non-cholecystectomized group (24). In addition, the study also observed a positive relationship between the female gender and CRC. Moreover, a previous study confirmed that patients who performed cholecystectomy presented a 108% higher risk of developing CRC than the common population. The male and female patients who underwent cholecystectomy were reported to have a 74 and 154% higher risk of CRC, respectively (23). Furthermore, age was also regarded as a risk factor for gastrointestinal cancers in patients with a history of cholecystectomy (23). The standardized incidence ratio (SIR) was highest in individuals between 40 and 49 years old, followed by those in their ages more than 80 years old (23). The reported median duration from cholecystectomy to the diagnosis of CRC was about 5–15 years or more (34, 35). Additionally, the right colon is more prone to be affected by cholecystectomy. Giovaimucci et al. (34) believed that the proximal and distal colon is related to the different sources of embryos, and they present different sensitivity to carcinogens. The right colon is more sensitive to bile acids, probably due to the high amount of stool fluid in the right colon. Thomas et al. (36) believed that the concentration of secondary bile acids and the activity of 7α-dehydroxylase were higher in the right colon than in the left colon, and there were obvious differences in bile acid metabolism, leading to the susceptibility of CRC in the right colon after cholecystectomy.

## Cholecystectomy promotes the development of colorectal cancer by alternation of bile acid metabolism

### Synthesis, transport and metabolism of bile acids

Bile acids are the main components of bile and are synthesized in the hepatocytes via cytochrome P450-mediated oxidation of cholesterol (37, 38). This process takes place through two biosynthetic pathways: the “classical” and an “alternative” pathway (39). During the “classical” pathway, three cholesterol hydroxylase enzymes cholesterol 7α-hydroxylase (CYP7A1), sterol 12α-hydroxylase (CYP8B1) and mitochondrial sterol 27-hydroxylase (CYP27A1) produce the primary bile acids cholic acid (CA) and chenodeoxycholic acid (CDCA) (40, 41). The “alternative” pathway produces CDCA via the hydroxylation of the cholesterol side chain by CYP27A1, and the oxysterol intermediates are then formed by 7α-hydroxylation by CYP7B141 (40). Bile acids can be divided into free bile acids and conjugated bile acids according to their structures. Free bile acids include CA, deoxycholic acid (DCA), CDCA, and lithocholic acid (LCA). The free bile acids are combined with glycine or taurine respectively to form various corresponding conjugated bile acids, including glycocholic acid, taurocholic acid, glycochenodeoxycholic acid and taurochenodeoxycholic acid. Conjugated bile acids are more water-soluble and generally exist in the body as sodium salts, which are more stable than free bile acids. In addition, bile acids can be divided into primary and secondary bile acids according to their sources. Primary bile acids are synthesized directly from cholesterol in hepatocytes including CA and CDCA, while secondary bile acids are formed when primary bile acids are secreted into the intestine and undergo 7-α-hydroxylation by intestinal bacteria including DCA, LCA, ursodeoxycholic acid (UDCA), and tauroursodeoxycholic acid (TUDC). Bile acids are secreted through the tubular membrane into the bile and stored in the gallbladder. After the animal eats, the duodenum secretes cholecystokinin, which stimulates gallbladder contraction, thereby releasing bile acids into the small intestine. In the small intestine, conjugated bile acids specifically activate pancreatic lipases and enhance fat-soluble vitamins solubilization by creating mixed micelles of dietary lipids, sterols, and fat-soluble vitamins. Finally, about 95% bile acids are reabsorbed in the ileum and returned to the liver via the portal vein. However, approximately 5% bile acids escaping from intestinal reabsorption enter the colon, where they are further converted to secondary, more hydrophilic bile acids by the intestinal flora (40, 42, 43).

### Cholecystectomy changes metabolism of bile acids

Under normal conditions, the gallbladder controls the rate and flow of bile into the intestine and enterohepatic circulation of bile acids, which plays a key role in regulating physiological homeostasis (44). However, the rhythm of bile acids entering the intestine is altered after cholecystectomy, which leads to metabolic disorders. The normal bile acid pool is the total amount of bile acids in the enterohepatic circulation, which is about 3 g and consists of 50% CA, 30% CDCA, 20% DCA and very small amounts of other bile acids. Previous studies showed that the bile acid reabsorption and enterohepatic circulation increase due to the sphincter of Oddi disorders after cholecystectomy (45, 46). However, there is also small number of studies finding that the bile acid pool decreases or remains unchanged after cholecystectomy. For example, a previous study observed that the bile acid pool decreased by about 16% three months after cholecystectomy (47). Two animal experiments showed that the total bile acid pool decreased by about 40% two weeks after cholecystectomy, as well as the reduction of circadian rhythm (48, 49). In addition, the total amount of bile acids remained essentially unchanged after five to eight years of cholecystectomy (46). The above findings suggest that the size of bile acid pool decreases in the short-term outcome after cholecystectomy, but there is no significant effect on the long-term outcome. Moreover, it has been confirmed that cholecystectomy increases the bacterial uncoupling and dehydroxylation of bile acids, thereby leading to the high proportion of secondary bile acids (44). Zhang et al. (48) demonstrated that the contents of DCA, LCA and their binding products with taurine were significantly increased in the ileum of mice after cholecystectomy, together with the increasing of fecal bile acid.

### Carcinogenic effects of secondary bile acids on colorectal cancer

Numerous experimental studies have confirmed the tumorigenic potential of bile acids, particularly the secondary bile acids DCA and lesser extent of the LCA (50–55). It is important to note that the bile acids are usually considered as tumor promoters rather than tumor inducers, because the changed bile acid concentrations depend on exposure to carcinogenic chemicals or genetic susceptibility (56). The carcinogenic effects of secondary bile acids on CRC are summarized in Table 1.

**TABLE 1:** The carcinogenic effects of secondary bile acids on CRC.

Authors (references)	Published year	Country	Cells/Animals	Types of bile acid	Effects, genes, and/or pathways
Cheng et al. (17)	2005	United States	SNU-C4 and H508	GDCA, DCA	Bile acids enhances CHRM3-dependent cell proliferation by transactivation of EGFR
Pai et al. (57)	2004	United States	SW480, LoVo	DCA	DCA promotes cell growth and invasiveness by activation of β-catenin signaling
Milovic et al. (59)	2002	Germany	Caco-2, HT-29	DC	DC promotes cell proliferation at low-dose, while induces apoptosis at high dose
Fu et al. (60)	2019	United States	Murine mice, HCT116, Caco2, HT29	DCA	DCA promotes cancer stem cell proliferation
Sorrentino et al. (64)	2020	Switzerland	Murine mice	DCA, LCA	Bile acids activate intestinal stem cells and epithelial regeneration via TGR5
Qiao et al. (66)	2001	United States	HCT116	DCA	DCA presents a dual role in apoptosis via the ERK/MAPK pathway
Farhana et al. (73)	2016	United States	HCoEpiC	DCA, LCA	Bile acids promote colon stemness in colonic epithelial cells via CHRM3 and Wnt/β-catenin signaling
Qiao et al. (80)	2001	United States	HCT116	DCA	DCA downregulates p53 via stimulating the ERK signaling pathway
Hu et al. (81)	2015	United States	HCT116, HT29	DCA, LCA	Bile acids promote Nur77-mediated cell proliferation and apoptosis
Lechner et al. (89)	2002	Germany	HT-29	DCA	DCA causes oxidative stress and increases TR level
Halvorsen et al. (91)	2000	Norway	CaCo-2	LCA	LCA increases cell invasion through promoting MMP-2 secretion
Nguyen et al. (92)	2017	Korea	HCT116	LCA	LCA induces expression of IL-8 by activating ERK1/2 MAPK and inhibiting STAT3
Centuori et al. (96)	2016	United States	HT-29	DCA	DCA promotes cell viability via activation of EGFR-MAPK pathway
Nagathihalli et al. (97)	2014	United States	HCT116, HCA-7	DCA	DCA regulates cell cycle by activation of EGFR, MAPK and STAT3 signaling
Zhu et al. (98)	2012	United States	HT-29, Caco-2, HCA7, HCT116	DCA	DCA promotes proliferation and invasiveness by activation of COX-2 signaling
Li et al. (100)	2003	Japan	HCT116, DLD-1, SW620	DCA	DCA upregulates EPHA2 via activation of ERK 1/2 cascade
Milovic et al. (101)	2001	Germany	Caco-2	DCA	DCA promotes cell migration via PKC
Debruyne et al. (102)	2002	Debruyne	HCT-8/E11, SRC transformed PCmsrc cells	DCA, LCA, CDCA	Bile acids stimulate cell invasion and haptotaxis via RhoA/Rho-kinase pathway and signaling cascades (PKC, MAPK, and COX-2, etc.)
Lee et al. (103)	2010	Korea	HM3	DCA	DCA upregulates MUC2 transcription via activation of EGFR/PKC/Ras/Raf-1/MEK1/ERK/CREB, PI3K/Akt/IKKB/NF-κB and p38/MSK1/CREB and inactivation of JNK/c-Jun/AP-1 pathway
Lee et al. (105)	2004	Korea	HT-29	DCA	DCA induces IL-8 expression and exerts anti-apoptotic effect via activation of NF-κB
Song et al. (106)	2005	United States	LiM6	DCA, LCA, CDCA	DCA upregulates MUC2 transcription via MAPK, PKC-dependent activation of AP-1
Baek et al. (107)	2010	Korea	HT29 and SW620	LCA	LCA enhances cell invasiveness by increasing expression of uPAR via activation of ERK1/2 and AP-1 pathway

CRC, colorectal cancer; GDCA, glycodeoxycholic acid; DCA, deoxycholic acid; DC, deoxycholic; LCA, lithocholic acid; CHRM3, cholinergic receptor muscarinic 3; EGFR, epidermal growth factor receptor; TGR5, G protein-coupled bile acid receptor 1; ERK, extracellular signal regulated kinases; MAPK, mitogen activated protein kinase; TR, thioredoxin reductase; MMP2, matrix metalloproteinase 2; IL, interleukin; STAT, signal transduction and transcriptional activator; COX-2, cyclooxygenase 2; EPHA2, EPH receptor A2; PKC, protein kinase C; CREB, cAMP response element binding protein; PI3K, phosphoInositide-3 kinase; IKKB, Ikappa B; NF-κB, nuclear factor kappa-B; MSK1, mitogen and stress-activated protein kinase 1; AP-1, activated protein-1; JNK, c-jun N-terminal kinase; MUC2, mucin 2, oligomeric mucus/gel-forming; uPAR, urokinase-type plasminogen activator receptor.

DCA has been reported to enhance colonic epithelial and colon cancer cell proliferation and/or invasiveness (57–60), promotes dysplasia (61), and disrupts the cell monolayer integrity of intestinal cancer and precancerous cells, increases the production of pro-inflammatory cytokines (51, 62), promotes cell cycle arrest (63), and activate intestinal stem cells and epithelial regeneration (64). In addition, DCA has been demonstrated to inhibit wound healing in wounded colonic epithelial monolayers by impairing cell migration ability (65). Interestingly, DCA exerts pro-apoptotic and anti-apoptotic effects on colon cells (66). It has been revealed that DCA could promote transition from adenoma to carcinoma and resist apoptosis (67), and also induce epithelial-mesenchymal transition (EMT) process, increase vasculogenic mimicry (VM) formation (68). Furthermore, DCA could help cancer cells to escape immune surveillance (69). Besides, DCA was found to cause a redistribution of cholesterol and decrease the fluidity of the membranes (70). As well, previous studies have confirmed that DCA could be converted into a powerful carcinogen 3-methylcholanthrene (3-MC) (71), and also regulate cell junction and increase intestinal permeability (72). Besides, DCA/LCA could increase drug resistance and induce colon carcinogenesis (73).

With respect to the molecular mechanism, DCA and/or LCA was reported to induce expressions of cyclooxygenase (COX)-2 promoter (74) by transactivation of the epidermal growth factor receptor (EGFR) in HCT116, H508 and SNU-C4 human colon cancer cell lines (17, 75), promote the stable and translocated pronucellin entering the nucleus and stimulate the expression of uPA, urokinase-type plasminogen activator receptor (uPAR) and cyclin D1 in SW480 and LoVo cells (57), activate muscarinic receptor (MR) in H508 human colon cancer cells (76), increase the expression of matrix metalloproteases (MMPs) in H508 cells (77), inhibit the effect of microRNA (miR)-199a-5p and/or promote the expression of CDK2 associated cullin domain 1 (CAC1) in HCT-8 cells (78). These above processes were associated with proliferation and invasion of DCA. For the anti-apoptotic characteristics, DCA was shown to upregulate the expression of X-linked inhibitor of apoptosis protein (XIAP) in normal intestinal epithelial cells (IEC-6), while downregulate the expression of p53 in HCT116 cells (79, 80). It is noted that Hu et al. suggested that DCA and/or LCA presented a dual role in modulating cell survival and death by regulating expression of Nur77 and intracellular location in HCT116 and HT29 colon cancer cells (81). Likewise, DCA was revealed to decrease the expression of human leukocyte antigen (HLA) class I antigens on the surface of HT29, SK-CO-l and SW1116 cells to help cancer cells to escape immune surveillance (69). As well, DCA was also described to prompt colonic epithelial cells HCoEpiC into becoming cancer stem cells (CSCs) (73), and form aberrant crypt foci (ACF) and high-grade dysplasia in AKR/J mice (82). Besides, DCA endorses the recruitment of tumor-associated macrophages (TAM) (83), decrease the levels of secretory antibodies of the type IgA (sIgA) and promotes polarization of M2 macrophages in APC^min/+^ mice (51). Interestingly, DCA has also been reported to cause genomic instability including heteroploidy, intrachromosomal instability and gene point mutations (84). The genomic instability appears via several mechanisms, comprising DNA oxidative damage, mitochondria damage, endoplasmic reticulum damage, micronucleus rate increase, disruption of mitosis, and mutations of chromosome aneuploidy (85, 86). DCA-induced DNA oxidative damage is caused after long-term exposure to high concentrations of nitro DCA and oxidation, which can induce apoptosis or DNA damage. Long-term DNA damage leads to mutation and natural selection of mutant cells, and ultimately promotes the development of cancer cells (87). Moreover, DCA can cause abnormal functions of some DNA mismatch repair enzymes by inducing mutations, such as adenomatous polyposis coli (APC) and tumor protein p53 (TP53). Subsequently, the dysfunction of DNA mismatch repair causes genome microsatellite instability (88). Long-term exposure to a high concentration of secondary bile acids can generate reactive oxygen species (ROS), induce oxidative stress (89), active nitrogen species, and cause DNA damage in intestinal epithelial cells, leading to genomic instability and increase gene mutations (90). In contrast, LCA has been reported to promote CRC via promoting expression of MMP-2 in CaCo-2 cells (91), interleukin (IL)-8 in HCT116 cells (92), ATP binding cassette subfamily B member 1 (ABCB1), ATP binding cassette subfamily G member 2 (ABCG2) in HCoEpiC cells (73), and miR-21, and inhibition of PTEN in HCT116 cells (93). As well, LCA induces DNA single-strand breaks (94) and inhibits mammalian DNA polymerase β in rat colon epithelial cells (95).

Several signaling pathways have been reported to be involved in the tumor-promoting effect of DCA on CRC. For example, DCA promotes CRC by activation of EGFR-mitogen activated protein kinase (MAPK), and induction of calcium in HT-29 cells (96) and signal transduction and transcriptional activator (STAT) 3 signaling pathways in HCT116 and HCA-7 cells (97). DCR facilitates proliferation and invasiveness through COX-2 in HT-29, Caco-2, HCA7, and HCT116 cells (98) and/or COX-2/prostaglandin E2 (PGE2) signaling pathway in human colonic fibroblasts CCD-18Co cells (99). In addition, DCA and/or LCA has been demonstrated to promote CRC by regulating Wnt/β-catenin signaling in SW480, LoVo, or HCoEpiC cells (57, 73), activation of extracellular signal regulated kinases (ERK) 1/2 cascade in HCT116, DLD-1, and SW620 cells (100), protein kinase C (PKC) in Caco-2 cells (101), RhoA/Rho-kinase pathway in HCT-8/E11 and SRC transformed PCmsrc cells (102), EGFR/PKC/Ras/ERK/cAMP response element binding protein (CREB), phosphoInositide-3 kinase (PI3K)/Akt/IkappaB (IKKB)/nuclear factor kappa-B (NF-κB) and p38/mitogen and stress-activated protein kinase 1 (MSK1)/CREB pathways and inactivates c-jun N-terminal kinase (JNK)/c-Jun/activated protein-1 (AP-1) pathway (103) and p53 pathway (80). Moreover, DCA activates JNK 1/2, and AKT signaling pathways that result in selective resistance to apoptosis, angiogenesis, proliferation and oxidative stress (40, 104). Furthermore, DCA is also reported to activate anti-apoptotic effect of NF-κB and induces IL-8 (105) and to upregulate MUC2 transcription via MAPK, PKC-dependent activation of AP-1 pathway in LiM6 cells (106). Meanwhile, LCA induces expression of uPAR and increases cell invasiveness via activation of ERK1/2 MAPK and AP-1 pathway in HT29 and SW620 cells (107) and inactivation of STAT3 and Src/EGFR pathways in HCT116 cells (92, 108, 109).

## Cholecystectomy promotes the development of colorectal cancer by changing the gut microbiota

### Gut microbiota and colorectal cancer

The intestinal flora is a great deal number and diversity of microbial species, which is the most significant micro-ecosystem in the human body. It has been estimated that approximately more than 500 species of bacteria from 30 genera exist in healthy adult intestines (110). These bacteria are composed of aerobes, facultative anaerobes and anaerobes, and most of them are obligate anaerobes or facultative anaerobes. Among the bacteria, 90% of the intestinal flora is *Bacteroidetes* and *Firmicutes* (111). The intestinal flora is a significant contributor in several physiological activities, such as food residue metabolism, micronutrient synthesis, primary bile acid metabolism, secondary bile acid synthesis, and immune response regulation (112). In addition, these bacteria is able to establish a biological barrier in the gut via space-occupying effect, nutrient competition, and some secreted metabolites (113), which can decrease low-grade inflammatory response in the body and maintain the integrity of the intestinal wall. Motivating the intestine to create an effective immune defense system can modulate the absorption and conversion of sugar and fat in the intestinal tract, subsequently ameliorate glucose tolerance and oxidative stress, and lower blood glucose (114). Therefore, the homeostasis of gut microbiota plays significant roles in maintaining human health (115–118). However, dysbiosis of intestinal flora is involved in a wide range of human diseases. A large of human and animal experiments has confirmed that the dysbiosis of gut microbiota shows cancer-promoting effects on gastrointestinal carcinogenesis, especially CRC (119–126).

### Cholecystectomy and gut microbiota

It has been well-acknowledged that cholecystectomy induces tremendous changes in the composition and function of the gut microbiota. For example, previous studies confirmed that after cholecystectomy, the number of *Bifidobacteria* and *Lactobacillus* was significantly decreased, while the number of *Enterococcus*, *Oscillospira*, *Escherichia coli*, *Bacteroidaceae* and *Bacteroidetes* was significantly increased (127–131). A previous study demonstrated that 1 mmol/L of DCA can effectively inhibit the growth of *Clostridium perfringens*, *Bacteroides fragilis*, *Lactobacillus* and *Bifidobacterium* in the intestinal tract (132). In addition, Cao et al. found that DCA significantly upregulated the populations of opportunistic pathogens, including *Ruminococcus*, *Escherichia-Shigella*, *Desulfovibrio*, and *Dorea*. Moreover, they also confirmed that DCA significantly increased the levels of *Clostridium* and *Escherichia-Shigella*, but markedly decreased the abundance of *Lactobacillus_gasseri* and mostly butyrate-producing bacteria, such as *Clostridium leptum Lachnospiraceae bacterium* and *Eubaterium coprostanoligenes* (83). On the contrary, an animal research showed that the population level of *Bacteroides* was increased in the ceca of rats fed with DCA (133). The main potential mechanisms include imbalance of bile acid metabolism, cellular immune abnormality, acid-base imbalance, and activation of cancer-related pathways and induction of toxin, inflammation and oxidative stress.

### Imbalance of bile acid metabolism

Fibroblast growth factors (FGF) are cellular factors that are synthesized by the terminal epithelial cells of the ileum and are involved in the regulation of bile acid metabolism (134). The FGF19 or FGF15 is transported to the liver through the portal vein system to inhibit bile acid synthesis. A previous study showed that the levels of FGF19 mRNA in the epithelial tissues of the gallbladder were 250 times higher than that in the terminal ileal epithelium (135). After cholecystectomy, the balance of bile acid metabolism is disturbed as the expression of FGF19 decreases and the primary bile acid production increases, altering the bidirectional interaction between bile acid and intestinal flora (128). The continuous drainage of bile into the intestinal lumen continuously stimulates intestinal motility, which increases peristalsis and shortens the total intestinal transit time. The enterohepatic circulation of bile acid is accelerated and the production of secondary bile acids is increased. The hydrophobic nature of secondary bile acids increases their affinity for the phospholipid bilayer of the intestinal bacterial cell membrane, leading to cell membrane damage and bacterial lysis and death (70).

### Cellular immune abnormality

The mucosal epithelium of the gallbladder could synthesize surfactant protein D (SP-D) (136). The SP-D is excreted into the intestinal lumen with bile and facilitates the synthesis of intestinal T cells (137). Intestinal T cells are involved in the regulation of inflammatory responses in the intestine. After cholecystectomy, the lack of SP-D in the gallbladder drastically reduces the number of intestinal T cells and predisposes the intestinal tract to bacterial infection and dysbiosis (137). Gallbladder surface protein D can also inhibit the growth of *Lactobacilli* in the intestinal tract by directly binding to *Lactobacilli* to induce lysis of *Lactobacilli* (137). Although *Lactobacillus* is beneficial to the human body, its excessive growth can affect the growth of other bacteria in the intestinal tract, thus causing dysbiosis of the intestinal flora.

### Acid-base imbalance

Small intestinal fluid is weakly alkaline, with a pH value of 8.0–9.0. Normal bile is weakly acidic, and its pulsatile secretion helps to create a good intestinal microbiological environment and maintain a stable pH value in the intestine. After cholecystectomy, alkaline bile is continuously secreted, which affects the pH balance in the intestine. The optimal pH values for the growth of *Lactobacilli* and *Bifidobacteria* are 5.5–6.0 and 6.5–7.0, respectively (138). Therefore, the increase in pH in the intestine inhibits the growth of beneficial bacteria such as *Lactobacillus* and *Bifidobacterium*, leading to dysbiosis.

### Activation of cancer-related pathways and induction of toxin, inflammation and oxidative stress

Activation of Wnt/β-catenin pathway has been implicated in the development and progression of CRC (139–141). E-cadherin is a well-known tumor suppressor, which can exert its function through β-catenin (142). The correlation between gut bacteria and E-cadherin/β-catenin has been reported. For example, *Fusobacterium nucleatum* has been demonstrated to attach E-cadherin on epithelial cells via its toxic factor FadA adhesin and stimulate β-catenin signaling pathway, and subsequently induce the gene expression of Wnt pathway (143). Meanwhile, a recent study also confirmed that, in addition to the effect on alteration of gut microbiota, DCA could downregulate the expression of E-cadherin, and increase nuclear β-catenin expression, as well as initiation of the downstream Wnt signaling molecules (83). Furthermore, *Fusobacterium nucleatum* has been reported to release RNA into the host cell cytoplasm, which could be detected by cytosolic retinoic acid-inducible gene 1 (RIG-1), and then activate NF-κB pathway, ultimately induce the expression of inflammatory genes and oncogenes (144, 145). Besides, FadA has been illustrated to bind to vascular endothelial cadherin (VE-cadherin), causing VE-cadherin to relocate and then increasing the permeability of endothelial cells, which enables *Fusobacterium* and other bacteria species to enter into the blood stream (146). *Peptostreptococcus anaerobius* has been identified as a novel microbial promoter of intestinal inflammation and tumor (147, 148). A recent research found that *Peptostreptococcus anaerobius* could interact with toll-like receptors (TLR)-2 and TLR-4 to motivate the generation of reactive oxidative species (ROS), which can stimulate the biosynthesis of cholesterol, leading to colon cell proliferation and dysplasia in mice (149). Additionally, alternation of bile acid has been revealed to induce the growth of pro-inflammatory bacteria, such as *Mogibacterium* and *Sutterella*, which may cause DNA damage and inflammatory response (150). Chronic inflammation could then indorse the event of IBD-associated dysplasia and development of adenoma-carcinoma sequence (151, 152). It has been revealed that *Bacteroides fragilis* could release bacteroides fragilis toxin (BFT), which activates a pro-carcinogenic multi-step inflammatory cascade through IL-17R, NF-κB and STAT3 pathways in colon epithelial cells (153) and contributes the development of polyp-adenoma-CRC (150). *Escherichia coli*, *Bacteroides fragilis*, *Providencia ewing*, *Micromonospora*, and *Peptostreptococcus anaerobius* have been displayed to induce CRC by production of a genotoxin colibactin that could induce DNA damage (154, 155).

## Outcomes of combined bile acid applications

Although DCA and LCA present tumor-promoting effects, UDCA is a therapeutic bile acid and has been reported to have a chemopreventive effect based *in vitro* and *in vivo* (156–160). Recently, UDCA was demonstrated to reduce the risk for advanced colorectal adenoma (161, 162) and CRC (156, 163). In addition, UDCA could modulate the gut microbiome (162). UDCA can inhibit DCA-induced apoptosis via modulation of EGFR/Raf-1/ERK signaling in HCT116 cells (164). Moreover, co-treatment with low-dose celecoxib and UDCA reveals to decrease cell growth in HT-29 colon tumor cells (165). Besides, UDCA inhibits Ras mutations, wild-type Ras activation, and expression of COX-2 in azoxymethane (AOM)-induced colon cancer in rats (166). However, a previous study found that long-term administration of high-dose UDCA was associated with an increased risk of colorectal neoplasia in patients with ulcerative colitis (UC) and primary sclerosing cholangitis (PSC) (167). Currently, there are inadequate data to support the routine application of UDCA for chemoprevention of CRC, either in the common population or among individuals who are at higher risk for CRC.

## Conclusions and prospections

**FIGURE 1:**
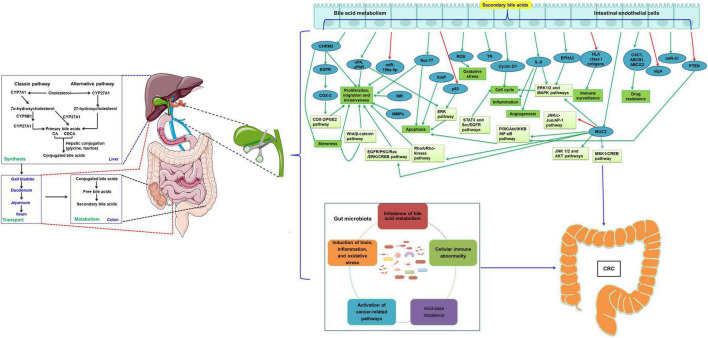
Cholecystectomy promotes the development of CRC by the alternation of bile acid metabolism and the gut microbiota. The green arrow indicates the levels are upregulated or the pathway is activated, while the red arrow indicates the levels are downregulated or the pathway is inactivated. CRC, colorectal cancer; CYP7A1, cholesterol 7α-hydroxylase; CYP8B1, sterol 12α-hydroxylase; CYP27A1, mitochondrial sterol 27-hydroxylase; CA, cholic acid; CDCA, chenodeoxycholic acid; COX-2, cyclooxygenase 2; EGFR, epidermal growth factor receptor; uPAR, urokinase-type plasminogen activator receptor; MR, muscarinic receptor; MMPs, matrix metalloproteinases; miR, microRNA; MUC2, mucin 2, oligomeric mucus/gel-forming; TR, thioredoxin reductase; IL, interleukin; EPHA2, EPH receptor A2; ABCB1, ATP binding cassette subfamily B member 1; ABCG2, ATP binding cassette subfamily G member 2; HLA, human leukocyte antigen; sIgA, secretory antibodies of the type IgA; XIAP, X-linked inhibitor of apoptosis protein; ROS, reactive oxygen species; PGE2, prostaglandin E2; ERK, extracellular signal regulated kinases; CREB, cAMP response element binding protein; PI3K, phosphoInositide-3 kinase; IKKB, Ikappa B; NF-κB, nuclear factor kappa-B; MAPK, mitogen activated protein kinase; STAT, signal transduction and transcriptional activator; PKC, protein kinase C; MSK1, mitogen and stress-activated protein kinase 1; AP-1, activated protein-1; JNK, c-jun N-terminal kinase.

There are many studies on the pathogenesis of CRC. In this paper, we reviewed the recent studies on the effects of cholecystectomy on CRC (Figure 1). The results show that cholecystectomy might promote the development of CRC by alteration of bile acid metabolism and the gut microbiota. The occurrence of CRC is related to changes in bile acid metabolism, the composition and function of the gut microbiota, and/or the interaction between the two factors. General surgeons should strictly grasp the indications of cholecystectomy. Cholecystectomy is necessary for acute and chronic cholecystitis, symptomatic cholelithiasis, biliary tract movement disorders, non-calculous cholecystitis, gallbladder tumors or polyps, and biliary pancreatitis. Gallbladders with good contractile function should be preserved as much as possible, not blindly removed. However, whether other physiological changes after cholecystectomy are associated with intestinal flora, affecting the occurrence and development of CRC, and whether there is a direct correlation between the carcinogenic effect of secondary bile acids and intestinal microorganisms after cholecystectomy are still unclear and need to be further investigated. With the continuous research on the pathogenesis of secondary bile acids-induced CRC, targeted therapies, including targeted bile acid metabolism and intestinal microflora regulation, may be promising treatment strategies for CRC.

## Data availability statement

The original contributions presented in this study are included in the article/supplementary material, further inquiries can be directed to the corresponding author.

## Author contributions

YS conceived the project. XJ and ZJ performed the research. QC and WS collected the background information. XJ, ZJ, QC, WS, and MJ drafted the manuscript. YS revised the manuscript. All authors approved the publication of the manuscript.
